# IHF Is Required for the Transcriptional Regulation of the *Desulfovibrio vulgaris* Hildenborough *orp* Operons

**DOI:** 10.1371/journal.pone.0086507

**Published:** 2014-01-21

**Authors:** Anouchka Fiévet, Eric Cascales, Odile Valette, Alain Dolla, Corinne Aubert

**Affiliations:** 1 Laboratoire de Chimie Bactérienne, Institut de Microbiologie de la Méditerranée, CNRS, Marseille, France; 2 Laboratoire d’Ingénierie des Systèmes Macromoléculaires, Institut de Microbiologie de la Méditerranée, CNRS, Marseille, France; Florida International University, United States of America

## Abstract

Transcriptional activation of σ^54^-dependent promoters is usually tightly regulated in response to environmental cues. The high abundance of potential σ^54^-dependent promoters in the anaerobe bacteria, *Desulfovibrio vulgaris* Hildenborough, reflects the high versatility of this bacteria suggesting that σ^54^ factor is the nexus of a large regulatory network. Understanding the key players of σ^54^-regulation in this organism is therefore essential to gain insights into the adaptation to anaerobiosis. Recently, the *D. vulgaris orp* genes, specifically found in anaerobe bacteria, have been shown to be transcribed by the RNA polymerase coupled to the σ^54^ alternative sigma factor. In this study, using *in vitro* binding experiments and *in vivo* reporter fusion assays in the *Escherichia coli* heterologous host, we showed that the expression of the divergent *orp* promoters is strongly dependent on the integration host factor IHF. Bioinformatic and mutational analysis coupled to reporter fusion activities and mobility shift assays identified two functional IHF binding site sequences located between the *orp1* and *orp2* promoters. We further determined that the *D. vulgaris* DVU0396 (IHFα) and DVU1864 (IHFβ) subunits are required to control the expression of the *orp* operons suggesting that they form a functionally active IHF heterodimer. Interestingly results obtained from the *in vivo* inactivation of DVU0396, which is required for *orp* operons transcription, suggest that several functionally IHF active homodimer or heterodimer are present in *D. vulgaris*.

## Introduction

*Desulfovibrio vulgaris* Hildenborough (DvH) is a well-studied sulfate-reducing bacteria (SRB). It is the first SRB for which the genome has been sequenced [1] and genetics and cellular tools have been recently developed [2]. It is an anaerobic microorganism that exhibits a mode of growth based on the reduction of sulphate as a terminal acceptor and thus is directly linked to natural global sulphur and carbon cycles [3]. These microorganisms have particularly important applications in biotechnology as they are involved in toxic heavy metal bioremediation, hydrogen sulfur decontamination in wastewater and biocorrosion [4]. Although SRBs are ubiquitous in anoxic habitats, they are found in a wide variety of ecological niches as illustrated by their metabolic versatility. Such environmental adaptations require stimuli perception and the subsequent modulation of the expression of relevant genes to optimize metabolism and physiology. Indeed, an unusually large number of response regulators are encoded within the DvH chromosome. Interestingly, 70 potential σ^54^ promoters have been identified by computational prediction [5], [6] suggesting that σ^54^ factor is the nexus of a large regulatory network in DvH. The alternative σ^54^ factor associates with the RNA polymerase holoenzyme to target specific genes. However, to proceed to transcription, the σ^54^-RNA polymerase complex requires the action of enhancer binding proteins (EBPs). EBPs usually bind to regulatory sequences upstream the σ^54^-dependent promoter [7], [8], [9], [10]. Efficient and functional interaction of σ^54^-RNA polymerase with a cognate EBP requires DNA bending, which is often facilitated by the integration host factor (IHF) protein, especially for promoters that lack flexible intrinsic bends [11], [12], [13], [14], [15]. In some cases, IHF has also been shown to recruit the σ^54^-RNA polymerase to its promoter [14]. IHF is involved in many cellular processes correlated with DNA functions such as replication, transcription, partitioning, transfer and packaging into phage particles [11], [16], [17]. Aside its role in specific transcriptional activation of σ^54^ promoters, IHF contributes to a wide variety of macromolecular processes and is recognized, with CRP, FNR, FIS, ArcA, Lrp and H-NS, as one of the seven global regulators in *Escherichia coli* [18], [19]. HU, H-NS, FIS and IHF are basic proteins involved in the organization of the DNA nucleoid architecture and are therefore considered as histone-like proteins [20]. In *E. coli* and related bacteria, IHF is a 20-kDa protein composed of two subunits, α and β. Although the αβ heterodimer is the predominant active form, the αα and ββ homodimers might be biologically active [21], [22], [23], [24].

In addition to the 70 putative σ^54^-dependent promoters, DvH genome analyses revealed the presence of 37 putative σ^54^-associated EBPs and several genes encoding IHFα and IHFβ subunits [25]. To date, little is known about the specific roles of σ^54^, the cognate EBPs and IHF in SRBs. Elucidation of their functions is thus essential to generate predictive model of the adaptation, stress responses of SRBs to environmental factors, and to develop effective SRB-based biotechnologies. In a recent study, we examined the function of the σ^54^-dependent transcriptional regulator DVU2106 in the regulation of the divergent *orp* operons in DvH, which encode proteins putatively involved in positioning the septum in cell division [6]. We showed that this EBP collaborates with the σ^54^-RNA polymerase to orchestrate the simultaneous expression of the *orp* genes cluster [6]. Here, we tested the contribution of IHF to this regulatory mechanism. In a reconstitution assay into the heterologous host *E. coli*, we found that IHF is required for efficient DVU2106-dependent activation of the σ^54^ *orp* promoters. Using electrophoretic mobility gel shift assays, the purified *E. coli* IHF protein was shown to bind to the *orp* promoters. We further localized IHF recognition sites between the σ^54^- and DVU2106-binding sequences and determined which of the three potential IHF binding sites were of physiological relevance using site-directed mutagenesis, reporter fusion activity and *in vitro* gel shifts. We identified DVU0396 (IHFα) and DVU1864 (IHFβ) subunits from DvH as the heterodimer functionally active to complement the transcription of *orp* operons in the IHF-deficient *E. coli* strain. Finally, we tested the role of the IHFα subunit on the transcription of the *orp* operons in DvH.

## Materials and Methods

### Bacterial Strains, Plasmids, Oligonucleotides and Growth Conditions

The bacterial strains and plasmids used in this study are listed in the Table S1. Custom oligonucleotides are listed in the Table S2. *E. coli* strains were routinely grown at 37°C in Luria-Bertani (LB) medium supplemented with the appropriate antibiotic when required (0.27 mM for ampicillin, 0.15 mM for chloramphenicol and 0.20 mM for kanamycin). Cultures of DvH were prepared in Postgate C medium [26] supplemented with 0.17 mM of kanamycin or 0.15 mM of thiamphenicol at 33°C in anaerobic conditions.

### DNA Manipulations

Standard protocols were used for cloning and transformations. All restriction endonucleases and DNA modification enzymes were purchased from New England Biolabs. For electrophoretic mobility shift assay, PCRs were performed using the Expand High Fidelity (Roche Diagnostics). Site-directed mutagenesis was performed using the PfuTurbo® or the PfuUltra™ High Fidelity DNA polymerases (Stratagene). Chromosomal DNA was purified using the Wizard Genomic DNA purification kit (Promega). Plasmid DNA was purified using the High Pure Isolation Plasmid Kit (Roche Diagnostics). PCR products and plasmid fragments were purified using MinElute kits (Qiagen).

### IHF Binding Site Directed Mutagenesis

Site-directed mutagenesis was performed using plasmids pT7.5-*porp2::lacZ*, pT7.5-*pDVU2106::lacZ* and pT7.5-*porp1::lacZ* as templates [6] and specific oligonucleotides carrying mismatches within the putative IHF binding sites. Oligonucleotide pairs 2105IHFmut-dir/2105IHFmut-rev, 2107IHFmut1-dir/2107IHFmut1-rev and 2107IHFmut2-dir/2107IHFmut2-rev were respectively used to modify the IHF binding sequences of the *orp2* promoter (5′-AAGATGTTTGATT to 5′-CCGATGTTTGGGT), the IHF binding sites 1 (5′-AATCAGAATAAAA to 5′-GGGCAGAATACCC) and 2 (5′-CATCACAAGCTCG to 5′-CGGGACAAGCCCC) of the *orp1* promoter. After PCR amplification of the whole plasmids, the methylated templates were eliminated by *Dpn*I digestion and mutated plasmids were recovered by transformation into *E. coli* DH5α competent cells. The accuracy of the mutagenesis has been verified by DNA sequencing.

### Expression Plasmids

For plasmids producing DvH IHFα, IHFβ and IHFα+β, the strong and constitutive *lpp* promoter was PCR amplified from *E. coli* K12 genomic DNA and cloned into the pOK12-2106 vector [6] downstream the *DVU2106* gene using the unique *EcoR*I and *Nde*I restriction sites, yielding pOK12-2106-lpp. The IHFα-encoding *DVU0396* or IHFβ-encoding *DVU1864* genes were PCR-amplified from DvH genomic DNA and cloned downstream the *lpp* promoter in pOK12-2106-lpp using the unique *Nde*I and *Not*I restriction sites to yield pOK12-2106-lpp-0386 and pOK12-2106-lpp-1864 respectively. For construction of the plasmid carrying both genes, *DVU0396* was PCR-amplified and cloned downstream DVU1864 using the *Not*I restriction site resulting in pOK12-2106-lpp-1864-0396.

### Strain Construction

The W3110 Δ*lacZ* Δ*ihfA E. coli* strain was constructed by P1 transduction from the BW25113 *ihfA::kan^R^* strain from the KEIO collection [27] and cassette excision using pCP20 as previously described [28] using W3110 Δ*lacZ* as recipient [29]. The DvH Δ*ihf*α mutant strain was constructed using previously described protocols [30], [31]. Briefly, the 455-pb fragment upstream the *DVU0396* gene was PCR-amplified using oligonucleotides DVU0395AscI_dir and CterDVU0395-SpeI and cloned into the *Asc*I and *Spe*I sites of plasmid pDel. The 460-pb fragment downstream *DVU0396* was PCR-amplified using oligonucleotides DVU0396MfeI_dir and DVU0396BglII_rev and cloned into *Mfe*I and *Bgl*II sites of the plasmid previously constructed to yield pDel*ihf*α. The accuracy of the cloned fragments was verified by restriction and DNA sequencing. The pDel*ihf*α plasmid was transferred into DvH cells by electroporation as following: DvH cells grown in PC medium were harvested at early stationary phase (OD_600_∼0.8–1) and washed twice with cold and degassed sterile MilliQ-water. Approximately 500 ng of the pDel*ihf*α plasmid were electroporated with a single 1.9 KV, 25 µF and 250 Ω pulse into DvH competent cells in BTX 1-mm gapped cuvette using a BTX Harvard Electro Cell Manipulator® ECM630 apparatus. Cells were recovered in 30 ml of PC medium, and the culture was supplemented with 20 µg/ml of thiamphenicol after 6 hours of incubation at 33°C. After 7 days of culture in the same conditions, the culture was diluted in PC medium supplemented with thiamphenicol. After three rounds of culture and dilution, individual colonies were recovered by plating on PE medium supplemented with thiamphenicol. The deletion of the *ihf*α gene was verified by colony-PCR.

### RNA Preparation

DvH was grown in three 80-ml independent cultures of DvH to an OD_595_∼0.4. The cells were harvested and resuspended in 200 µl of 10 mM Tris pH8. RNA was prepared by using the High Pure RNA kit (Roche Diagnostics) according to the manufacturer’s instructions with an additional DNase I digestion step.

### Real-time Quantitative PCR

10 µg of total extracted RNA were reverse transcribed by using random hexamers to yield cDNA. Real-time PCR was performed on a LightCyclerFastStart DNA Master^PLUS^ SYBR Green I Kit (Roche Diagnostics). cDNA was mixed with 0.25 µM of each primer and 2 µl of master mix in a 10 µl final volume. The primers pairs used to quantify the expression levels of the selected genes are shown in Table S2. PCR amplifications were carried out with one cycle at 95°C for 8 min, followed by up to 45 cycles at 95°C for 12 s, 60°C for 6 s and 72°C for 20 s. The specificities of accumulated products were verified by melting-curve analysis. The fluorescence derived from the incorporation of SYBR Green I into the double-stranded PCR products was measured at the end of each cycle to determine the amplification kinetics of each product. The fit points method described by the manufacturer was then applied to the results. Briefly, a horizontal noise band was determined as well as a log line fitting the exponential portion of the amplification curve. The intersections of these log lines with the horizontal noise lines identified the crossing points. These crossing points were determined for each gene in three conditions. Relative Expression Software Tool (REST) was used to calculate the relative expression of each gene in each condition using the 16S RNA gene as reference for normalization.

### β-galactosidase Assay

The activity of the β-galactosidase reporter was measured from mid-exponential growth phase cultures (OD_600_∼0.8) as previously described [32]. Each enzymatic assay was performed in triplicate with three independent biological samples. The values are reported as the average of these nine measurements.

### Purification of the *E. coli* IHF Heterodimer

*E. coli* MC4100 pTrc-IHF [33] was grown overnight in 10 ml of LB supplemented with kanamycin at 37°C with shaking and used to inoculate 1 liter of LB. This culture was grown until the OD_600_ reached 0.6, and 1 mM of IPTG was then added. After 3H of induction, the *E.coli* IHF heterodimer was extracted as described [33]. Briefly, cells were washed with 40 ml of Buffer E (25 mM Tris-HCl pH7.4, 1 mM EDTA, 3 mM β-mercaptoethanol, 100 mM NaCl, 1 mM PMSF and 10% glycerol). After centrifugation, cells were resuspended in 10 ml of Buffer E supplemented with DNase. Cells were broken by 3 passages in French-press and centrifugated at 45000 rpm during 1 H at 4°C. IHF heterodimer was then purified by two consecutive steps: immobilization onto a HiTrap Heparin HP column equilibrated in Buffer E and elution with a step gradient of KCl (0.2 M–2 M). The fractions containing IHF (0.8 to 1.2 M KCl) were pooled and diluted in Buffer A (25 mM Tris-HCl pH7.4, 1 mM EDTA, 3 mM β-mercaptoethanol and 10% of glycerol) to obtain a final KCl concentration of 100 mM, immobilized onto a HiTrap SP FF column and eluted with a step gradient of KCl. The fractions containing the IHF heterodimer (500–600 mM of KCl) were pooled and the buffer was exchanged against Buffer A. The final concentration of IHF was ∼1.5 mg/ml.

### Electrophoretic Mobility Shift Assay (EMSA)

^32^P-labeled probes were obtained by PCR amplification using a dNTP mix supplemented with [α-^32^P]-deoxyadenosine triphosphate and plasmid DNA as template. Labeled probes were column-purified to remove radioactive nucleotides (PCR Clean-up, Promega). For gel shift experiments, ^32^P-labeled probes were mixed with different concentration of purified IHF in a binding reaction mixture containing sonicated salmon sperm DNA (10 µg/mL [UltraPure™, Invitrogen]) and Bovine Serum Albumine (200 µg/mL [Fraction V, Sigma Aldrich]) in 40 mM Tris-HCl pH7.5, 50 mM NaCl, 40 mM NH_4_Cl, 5 mM MgCl_2_, 1 mM CaCl2, 8% glycerol, and 1 mM dithiothreitol (DTT). After incubation during 20 min at room temperature, DNA and DNA complexes were separated on a prerun 5,5% polyacrylamide (acrylamide:bis-acrylamide 29∶1) gel supplemented with 10% triethylene glycol [34] in Tris-Borate buffer. Gels were fixed in 10% trichloroacetic acid for 10 min and exposed to Kodak BioMax MR films.

## Results

### IHF is Required for *orp1* and *orp2* Transcription

**Figure 1:**
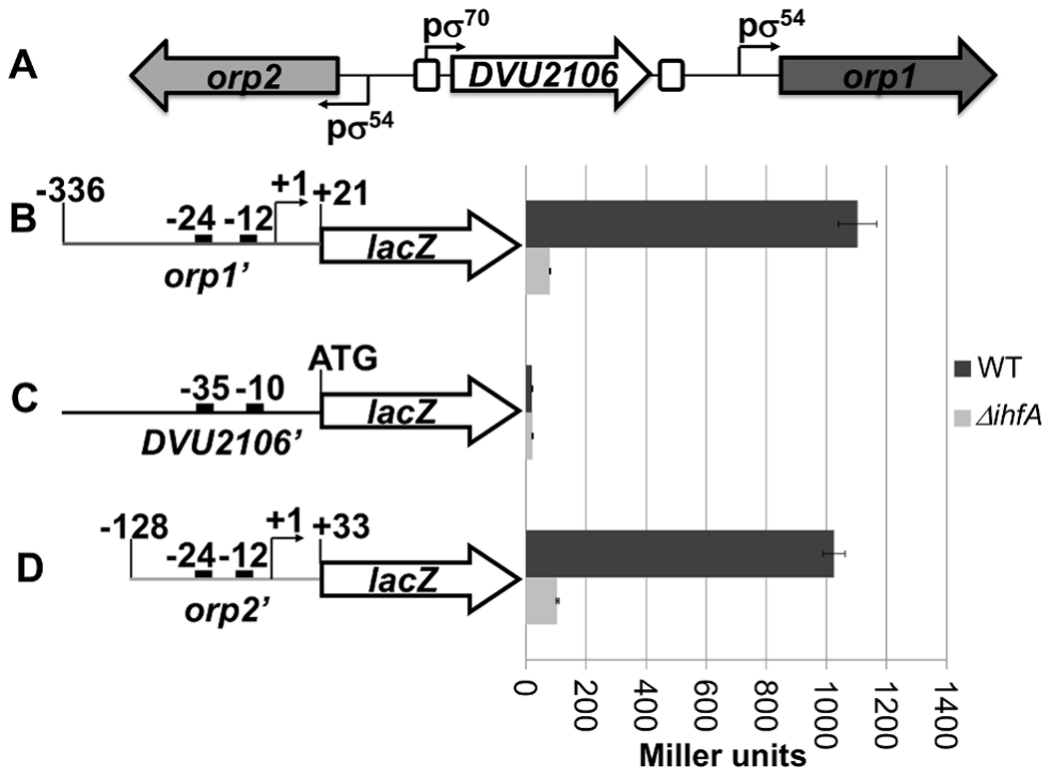
IHF is involved in the transcriptional activation of both operons *orp1* and *orp2*. (A) Schematic representation of the transcriptional elements of promoter regions of *orp1*, *DVU2106* and *orp2*. The positions of the σ^54^ and σ^70^ promoters are indicated by bent arrows and the DVU2106-binding sites are indicated by solid line boxes. The *lac*Z reporter fusions with the promoter regions of *orp1* (B), *DVU2106* (C) and *orp2* (D) are represented on the left. The transcript start sites are indicated by bent arrows. The positions of the −10 and −35 sequences of the σ^70^ promoter and the −12 and −24 sequences of the σ^54^ promoters are indicated by black rectangles. The activities measured in various backgrounds are shown on the right: *E.coli* wild-type strain (dark-grey) and *E.coli ΔihfA* strain (light-grey). The activity is the average of three independent measurements (the error bars show the standard deviations).

It was previously shown that the two divergent *orp* operons require the σ^54^ RNA polymerase and the cognate transcriptional EBP, DVU2106, to achieve transcription [6]. It is well described that DNA bending allowing contacts between the cognate activator and σ^54^-RNA polymerase to activate transcription is usually facilitated by the heterodimeric IHF protein. To test the contribution of IHF to the expression of these genes, the previously described *orp1*, *orp2* and *DVU2106* promoter-*lacZ* transcription fusions were introduced into the heterologous host *E. coli*. Expression of these promoter-*lacZ* fusions were examined in both IHF-proficient (wild-type) and IHF-deficient (Δ*ihfA*) strains that produced the transcriptional regulator DVU2106 from a compatible multicopy plasmid (Figure 1A–D). The expression of *DVU2106*, controlled by the σ^70^-RNA polymerase, was not influenced by the absence of IHF (Figure 1C). By contrast, the activities of the *orp1* and *orp2* promoters, under the control of the σ^54^-RNA polymerase, were significantly affected by the absence of IHF as the *orp1’::lacZ* and *orp2’::lacZ* expression levels decreased 10–12 fold compared to the wild-type strain producing IHF (Figure 1B and 1D). These results show that the *E. coli* IHF heterodimer can activate *orp* operons transcription and that IHF is required for the expression of these σ^54^-dependent operons.

### The *E. coli* IHF Protein Binds to the Promoter Regions of *orp1* and *orp2*

**Figure 2:**
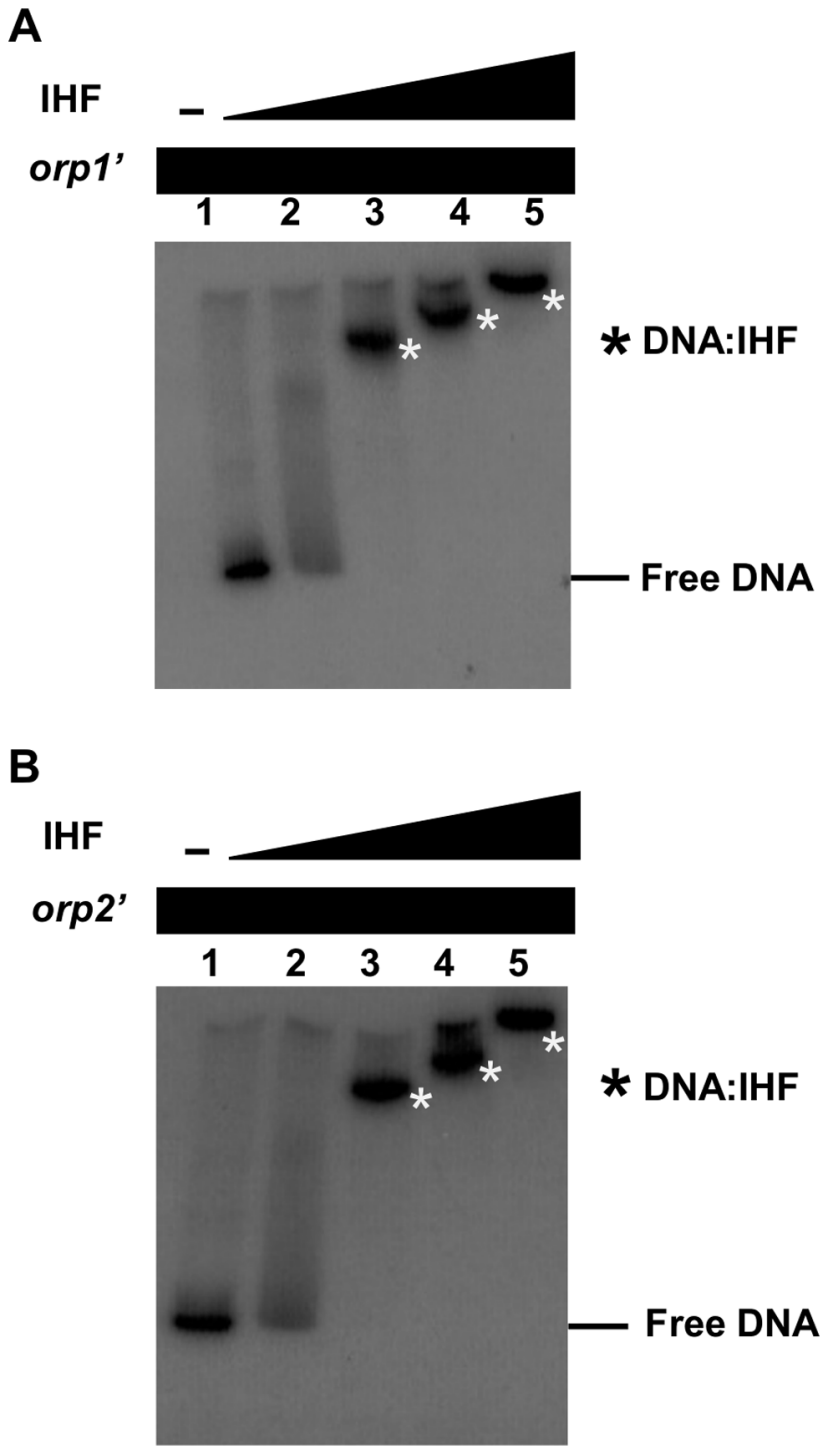
The integrator Host Factor (IHF) interacts with the promoter regions of *orp1* and *orp2*. Shown is the gel shift assay of the promoter regions of *orp1* (A) and *orp2* (B) using purified *E.coli* IHF heterodimer (lane 1, no protein; lane 2, 4 nM; lane 3, 8 nM; lane 4, 12 nM; lane 5, 16 nM).

To address whether IHF can bind to these promoters, we carried out electrophoretic mobility shift assays (EMSA). ^32^P-labeled *orp1* and *orp2* promoter fragments were incubated with increasing concentrations of purified *E. coli* IHF heterodimers. As shown in Figure 2, the two DNA fragments were specifically retarded in presence of ≥4 nM of IHF. These data demonstrate that the IHF protein interacts with the promoter regions of the *orp1* and *orp2* operons.

### Identification of the Functional IHF Binding Sites

**Figure 3:**
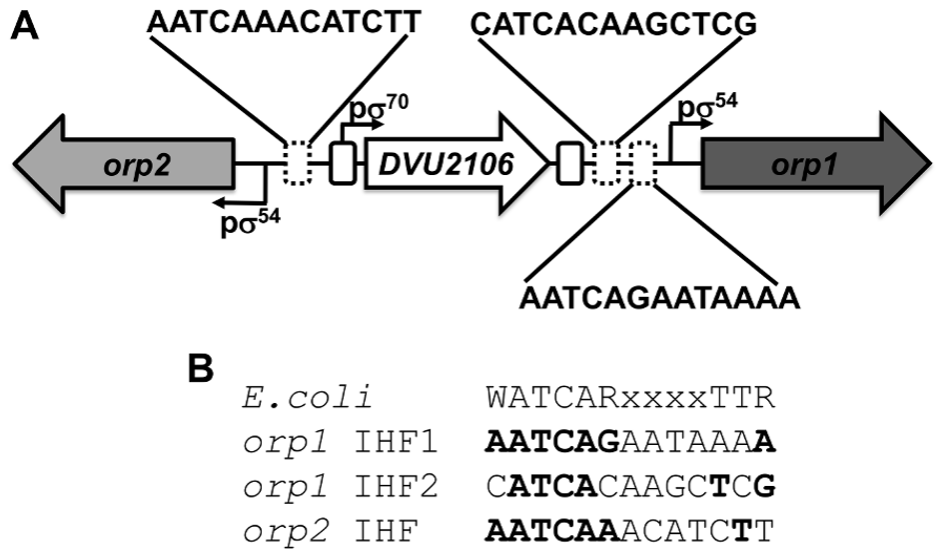
Sequence analyses of the *orp1* and *orp2* promoter regions. (A) The σ^54^ and σ^70^ promoters of the ORP system are indicated by bent arrows. The solid-line boxes indicate the palindromic DVU2106-binding sites. The dashed boxes represent the three putative IHF-binding sequences identified by comparison to the consensus sequence of the *E.coli* IHF-binding site (5′-WATCARxxxxTTR-3′). (B) DNA sequence alignment between the consensus sequence of *E.coli* IHF-binding site and each putative IHF-binding sequence of *orp1* and *orp2* promoter regions. The sequence identity between the consensus sequence of the *E.coli* IHF-binding site and the different *orp* putative IHF-binding site are indicated in bold.

*In silico* analyses of the intergenic region of the *orp* cluster using the Virtual Footprint program (http://prodoric.tu-bs.de/vfp/vfp_promoter.php) suggested the presence of two potential IHF binding sites within the *orp1* promoter (called hereafter IHF1 and IHF2) located at positions −42 and −79, respectively, from the transcriptional start site of *orp1* (Figures 3 and S1) and one potential binding site for IHF within the *orp2* promoter, located at position −43 from the transcriptional start site of *orp2* (Figures 3 and S1). The *orp1* IHF1 site (**AATCAG** AATAA**A** **)** [conserved nucleotides are in bold]) has 78% similarity with the reported *E. coli* consensus sequence with the conserved nucleotides mainly located at the 5′ extremity of the sequence whereas the *orp1* IHF2 site (C**ATCA**CAAGC**T**C**G** ) shares less similarity (67%) but better distributed throughout the sequence (Figures S1 and S3). The *orp2* potential IHF site (**AATCAA** ACATC**T**T) has 78% similarity with the reported *E. coli* consensus sequence. Regarding the relative position of these IHF binding sites compared to those of σ^54^ and of DVU2106, the *orp1* IHF1 (−42) and IHF2 (−79) sites are located upstream the σ^54^-binding sequence (position −13) and downstream the EBP binding site (position −131) (Figure S1). The *orp2* IHF binding site (−43) is located upstream the σ^54^-binding sequence (position −11) and downstream the EBP binding site (position −88) (Figure S1). Therefore, the locations of the three potential IHF binding sites are centred between σ^54^- and DVU2106-binding sequences.

**Figure 4:**
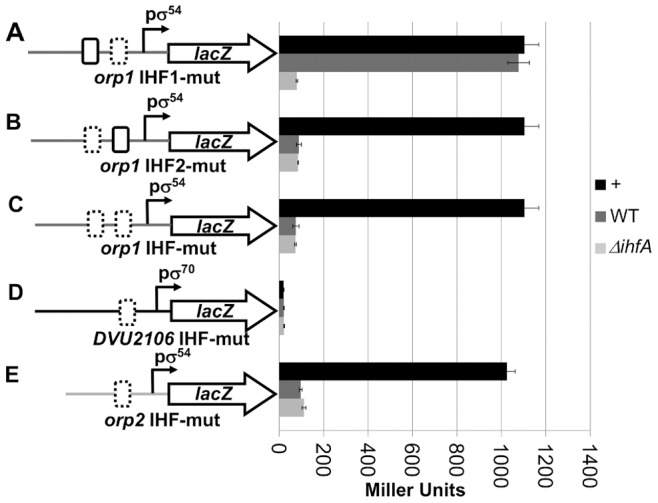
*orp1* IHF-2 and *orp2* IHF sites are involved in transcriptional activation of *orp1* and *orp2*, respectively. The *lac*Z reporter fusions with the promoter regions of (A) *orp1* IHF1-mut, mutated for the putative IHF-binding site 1, (B) *orp1* IHF2-mut, mutated for the putative IHF-binding site 2, (C) *orp1* IHF-mut, mutated for both IHF-binding sites, (D) *DVU2106* IHF-mut, mutated for putative IHF-binding site of *orp2* promoter and (E) *orp2* IHF-mut, mutated for the putative IHF-binding site are represented on the left. The nature of these mutations is described in materials and methods and in figure S4. Promoters σ^54^ and σ^70^ are indicated by bent arrows. The putative IHF-binding sites are indicated by boxes: solid-line boxes for wild-type sites and dashed boxes for mutated sites. The activities measured in various backgrounds are shown on the right: *E.coli* wild-type strain (dark-gray) and *E.coli* Δ*ihfA* strain (light-gray). The measures shown in black and indicated by (+) correspond to the β-galactosidase activity observed in a wild-type *E.coli* strain carrying the fusion between wild-type promoters and *lacZ*. The activity is the average of three independent measurements (the error bars show the standard deviations).

To determine the contribution of these IHF binding sites to the transcriptional activities of the *orp1* and *orp2* promoters, each of these consensus sites was altered by generating mutations at bases previously reported to be necessary for IHF binding [35] yielding *orp1* IHF1-mut, *orp1* IHF2-mut and *orp2*-IHF-mut and DVU2106-IHF-mut (*DVU2106* promoter region in which *orp2* IHF site was mutated) (Figure S2). These mutations were introduced into plasmids carrying promoters-*lacZ* transcription fusions. As done previously, expression of the wild-type and mutated promoter-*lacZ* fusions were compared in the IHF-proficient (wild-type) and IHF-deficient (Δ*ihfA*) strains that produced the transcriptional regulator DVU2106 from a compatible multicopy plasmid. The accumulation of β-galactosidase was measured and reported in Figure 4. The expression of *orp1* was not significantly affected by the mutation within the IHF1 site, as 98% of the β-galactosidase activity was retained compared to the IHF proficient strain with native *orp1’::lacZ* fusion (Figure 4A). By contrast, the β-galactosidase activity decreased to 8% of the activity of the wild-type promoter when the IHF2 site was altered (Figure 4B), a value similar to that of the wild-type reporter fusion in an IHF-deficient strain (compare Fig. 4B and Fig. 1B). When both sites were altered, the residual activity of the *orp1* promoter was comparable to that observed with the single IHF2 site mutation (Figure 4C). Regarding the expression from the *orp2* promoter, mutation of the potential IHF site led to a 10-fold decreased β-galactosidase activity compared to the wild-type reporter fusion (Figure 4E). Here again, this activity is comparable to that of the wild-type fusion in IHF-deficient cells (compare Fig. 4E and Fig. 1D). The expression of *DVU2106*, controlled by σ^70^-RNA polymerase, was not influenced by the absence of IHF protein and by the disruption of the *orp2* IHF site (Figure 4D). These results demonstrate the requirement of the *orp1* IHF2 and *orp2* IHF sites for the efficient transcription of the σ^54^-dependent *orp1* and *orp2* promoters, respectively. These results further suggest that theses sites could be targets for IHF binding.

**Figure 5:**
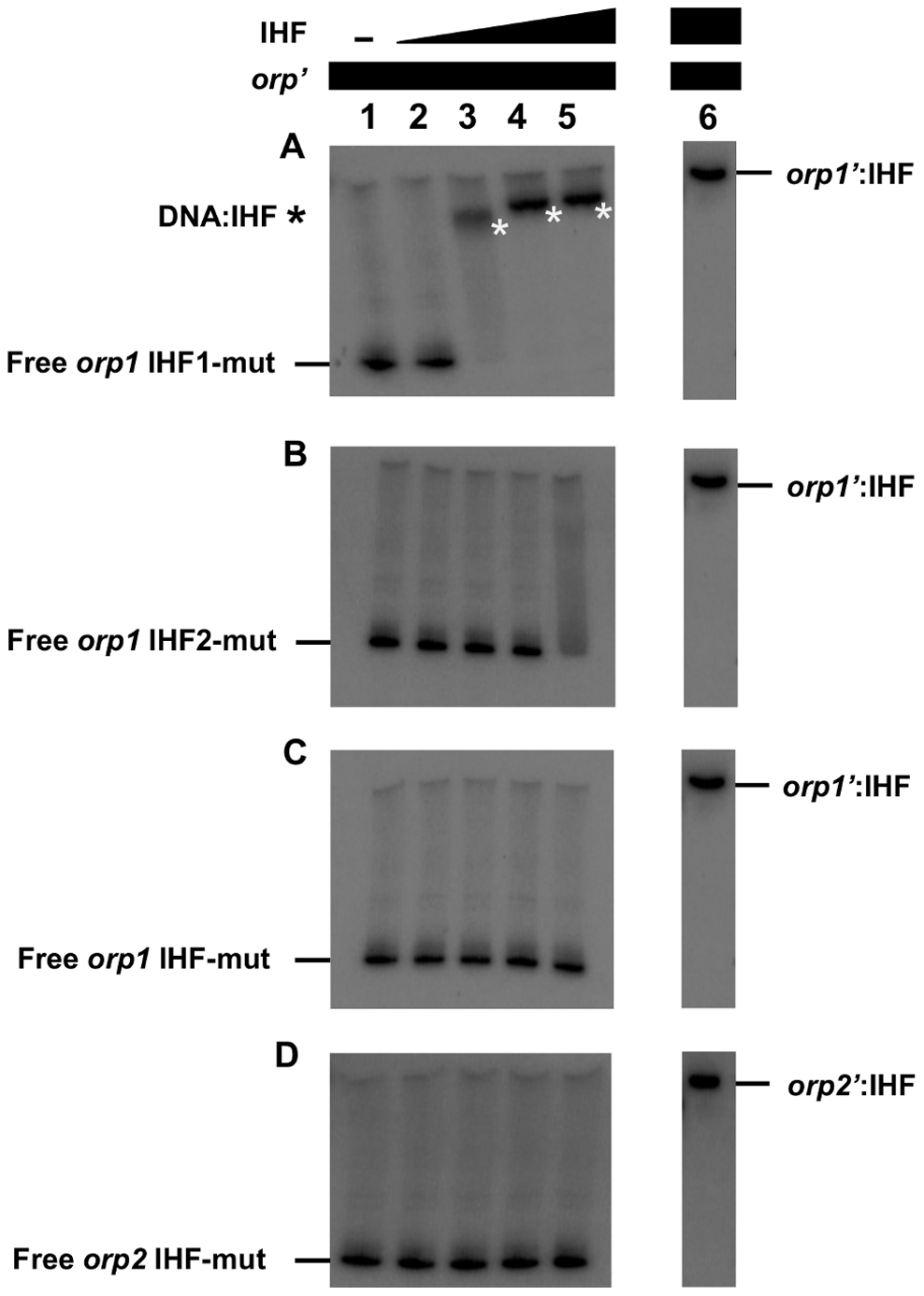
The integrator Host Factor (IHF) interacts with the site 2 of promoter region of *orp1* and the putative IHF binding site detected in *orp2*. Shown is the gel shift assays of the promoter regions of (A) *orp1* IHF1-mut, (B) *orp1* IHF2-mut, (C) *orp1* IHF-mut and (D) *orp2* IHF-mut, using purified *E.coli* IHF (lane 1, no protein; lane 2, 4 nM; lane 3, 8 nM; lane 4, 12 nM; lane 5, 16 nM). Lane 6 is from Figure 2 and corresponds to the gel shift assays of wild-type promoter fragments using 16 nM of purified *E.coli* IHF.

To address whether IHF binds to these putative IHF binding sites, we carried out EMSA experiments with increasing concentrations of the purified *E. coli* IHF protein and ^32^P-labeled *orp1* and *orp2* mutated promoters. In agreement with the β-galactosidase activities, figure 5 shows that disruption of the *orp1* IHF1 site did not prevent formation of the IHF-DNA complex (Figure 5A) whereas mutation within the IHF2 site (or within both IHF1 and IHF2 sites) abolished IHF binding (Figure 5B and 5C). Similarly, alteration of the *orp2* IHF site significantly prevented IHF binding to the *orp2* intergenic region (Figure 5D). It should be noted that 8 nM of IHF are needed for observing the formation of the IHF/*orp1* IHF2 complex when the *orp1* IHF1 site is altered (Figure 5A) whereas only 4 nM of IHF are necessary to form the same complex when *orp1* IHF1 is not altered (Figure 2A) suggesting that *orp1* IHF1 might promote binding of IHF on the IHF2 site.

Taken together, these results demonstrate that the *orp1* IHF2 site is required for IHF binding and *orp1* transcription and the *orp2* IHF site recruits IHF to activate *orp2* transcription. It is noteworthy that the purified IHF protein binds to sites centred between DVU2106 binding sites and σ^54^ promoters in both operons, a result compatible with IHF function in DNA bending to facilitate contacts between the distantly-located EBP and σ^54^-RNA polymerase.

### The DvH DVU0396-DVU1864 Heterodimer is Functionally Active to Stimulate the Transcription of the *orp* Operons

**Figure 6:**
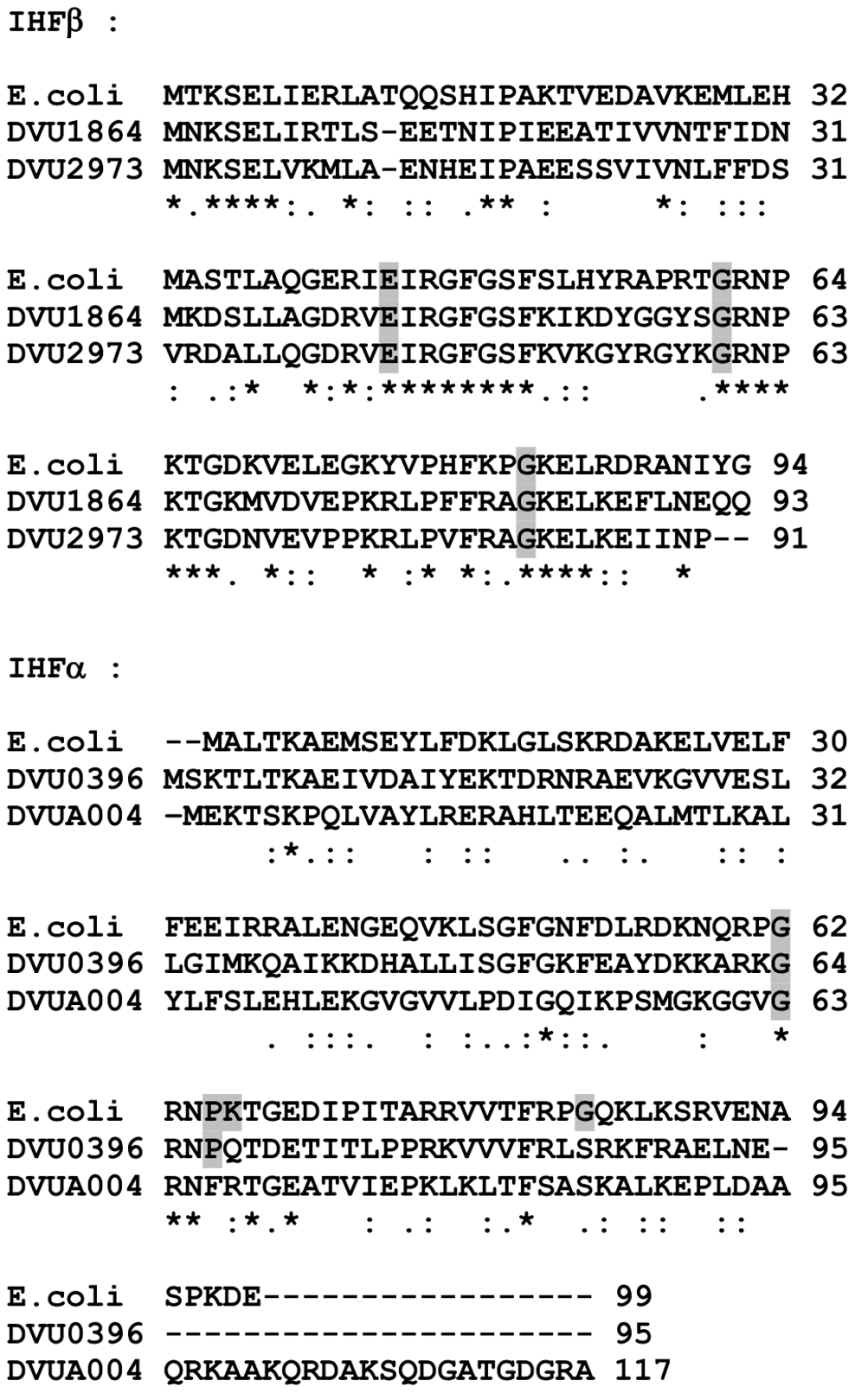
Protein sequence alignment of IHFβ and IHFα subunits of *E.coli* and DvH. (A) The figure shows the sequence of IHFβ from *E. coli* aligned with the sequences of the two putative IHFβ from DvH, DVU1864 and DVU2973. (B) The sequence of IHFα from *E.coli* was aligned with the sequences of the two putative IHFα, DVU0396 and DVUA004 from DvH using ClustalW (http://www.ebi.ac.uk). Highlighted residues correspond to crucial amino-acids for IHF function in *E. coli*.

IHF is usually described as a heterodimeric protein complex composed of the IHFα and IHFβ subunits encoded by the *ihfA* (*himA*) and *ihfB* (*himB*) genes. Genome analyses of various species known to encode active heterodimeric IHF revealed the presence of a single copy of genes encoding IHFα and IHFβ, found at distinct locations on the chromosome and subject to independent regulatory influences [36]. Surprisingly, two orthologs of *himB*, DVU1864 and DVU2973 that would encode IHFβ, are annotated on the DvH genome. Amino acid sequences of DVU1864 (called here β1) and DVU2973 (called here β2) are 65% identical together and respectively 42% and 45% identical to the *E. coli* IHFβ (Figure 6). By contrast, no gene encoding IHFα was annotated whereas six orthologs of *hup* genes encoding HU proteins were detected. Nevertheless, it has been previously proposed that two of the putative HU-encoding genes, *DVU0396* and *DVUA0004*, correspond to *himA* that encodes the IHFα protein [25]. Primary sequence comparison of DVU0396 and DVUA0004 with IHFα from *E. coli* revealed only 31% and 21% identity, respectively (Figure 6). More recently, results obtained from pull-down experiments using DVU1864 as bait identified DVU0396 as the preferential prey [37]. It should also be noted that *DVUA0004* is located on the endogenous plasmid of DvH, which is lost in our growth conditions.

**Figure 7:**
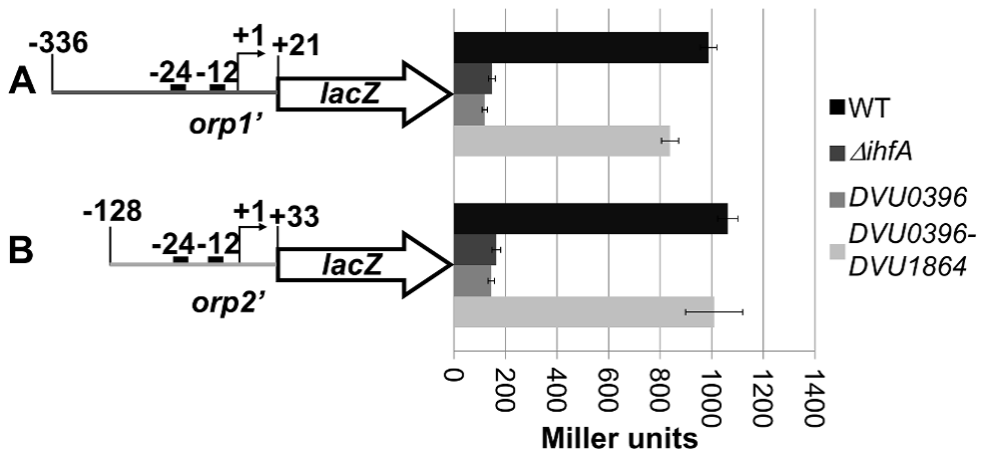
The heterodimer DVU0396-DVU1864 from DvH is involved in the transcriptional activation of *orp* operons. The *lac*Z reporter fusions with the promoter regions of *orp1* (A) and *orp2* (B) are represented on the left. The positions of the −12 and −24 sequences of the σ^54^ promoters are indicated by black rectangles. The transcript start sites are indicated by bent arrows. The activities measured in various backgrounds are shown on the right: *E.coli* wild-type strain (black), *E.coli* Δ*ihfA* strain (dark-grey), *E.coli* Δ*ihfA* strain producing DVU0396 (middle-grey) and *E.coli* Δ*ihfA* strain producing DVU0396-DVU1864 (light-grey). The activity is the average of three independent measurements (the error bars show the standard deviations).

In order to identify the active IHF from DvH, *orp1* and *orp2* promoter-*lacZ* transcription fusions were introduced into the IHFα-deficient *E. coli* (Δ*ihfA*) strain that produces the transcriptional regulator DVU2106 and the HU protein DVU0396 or both DVU0396 and DVU1864 from a compatible multicopy plasmid (Figure 7). Surprisingly, the construction producing DVU0396 was easily obtained in wild-type *E. coli* whereas the plasmid producing DVU0396 and DVU1864 was obtained after reducing the production of these two genes suggesting a toxic effect of the simultaneous production of both proteins. By contrast, no growth effect was observed when DVU0396 and DVU1864 are produced in the IHF-deficient *E. coli* strain (data not shown). Figure 7 shows that the β-galactosidase activities of the *orp1* and *orp2* reporter fusions were very low in the Δ*ihf*A strain, independently of the presence of the DVU0396 protein. However, the concomitant production of DVU0396 and DVU1864 in the Δ*ihfA* strain restored the activities of the *orp1* and *orp2* promoters to levels comparable to that observed in the wild-type strain. These results show that DVU0396 and DVU1864 are both required to activate *orp1* and *orp2* expression *in vivo* and further suggest that these two proteins may interact to assemble a functional heterodimer.

### Functional Analysis of the DvH *DVU0396 ihf*α Mutant

As shown above, the DVU0396 and DVU1864 proteins may interact to stimulate the transcription of the *orp* operons in the *in vivo* reconstitution approach using the heterologous host *E. coli.* The amino-acid sequences of the two putative IHFβ proteins encoded by *DVU1864* and *DVU2973* being 65% identical, one may suggest that both proteins may have redundant function. From these observations, and in order to determine whether IHF is physiologically required for the activation of the divergent *orp* operons in DvH, we deleted the *DVU03*9*6* gene on the DvH chromosome. Under anaerobic conditions and in lactate/sulfate medium, the deletion of *DVU0396* did not induce significant effects on DvH growth (final biomass and growth rate). The transcription levels of *orp1, orp2* and *DVU2106* were compared by qRT-PCR in the wild-type and the *DVU0396* mutant strains in both exponential and stationary phases. The expression ratio of *orp1*, *orp2*, and *DVU2106* [log2(absolute gene regulation) = −0.406, −0.404 and −0.805, respectively, using the wild-type strain as reference] showed that the absence of DVU0396 protein has no effect on *orp1* and *orp2* expression in DvH. Our further qRT-PCR analyses showed that the absence of DVU0396 is not compensated by an increasing expression of the genes encoding the IHFβ1 (DVU1864) or IHFβ2 (DVU2973) subunit [log2(absolute gene regulation) = 0.797 and 0.308 respectively, using the wild-type strain as reference]. Taken together these results indicate that the absence of DVU0396 is not compensated by the upregulation of genes encoding IHFβ subunits and does not affect the expression of the *orp* operons in DvH.

## Discussion

In most environments, microorganisms are under tough competition for available resources and have to perceive, integrate and respond to multiple signals pertaining to a variety of stresses that include limited nutrient availability, physicochemical stresses and pollutant toxicity. The ability to appropriately adapt to prevailing conditions usually involves complex regulatory mechanisms such as two-component systems and σ^54^-dependent promoters. In addition to the σ^54^ alternative factor, the σ^54^ pathway requires enhancer binding proteins and DNA bending. EBPs interact with an upstream activating sequence, and through DNA looping, which can be facilitated by IHF, contact the σ^54^-RNA polymerase. Interestingly, a large number of potential σ^54^-promoters and EBPs have been identified in DvH, an observation in accordance with the high metabolic versatility of this species [2]. To date nothing is known on the role of IHF in this bacterium and more particularly on its contribution to the σ^54^ regulatory mechanism. We recently showed that two gene operons, *orp1* and *orp2*, are regulated by σ^54^ and a cognate EBP, DVU2106 [6]. In this study, we have refined the regulatory mechanism underlying *orp* gene expression by demonstrating that IHF contributes to the regulation of these genes.

Using an *in vivo* reconstitution approach in *E. coli*, we showed that the activities of the divergently transcribed *orp1* and *orp2* operon promoters require the integration host factor IHF. Although the predominant role of IHF is to facilitate contacts between the EBP and the σ^54^-RNA polymerase, it was shown in *P. putida* that IHF is also important to recruit the σ^54^-RNA polymerase to its promoter [14]. The low expression of the *orp* operons measured in absence of IHF and the location of the IHF binding sites, located between the DVU2106 EBP- and the σ^54^-binding elements suggest a direct role of IHF to assist in the formation of direct contacts between DVU2106 and the RNA polymerase and/or to recruit the σ^54^-RNA polymerase to the appropriate site [38], [39], [40]. The activities of the *orp1* and *orp2* promoters were reduced 10–12 fold in absence of IHF or when IHF-binding boxes were altered, probably reflecting the limitations imposed by the absence of IHF-induced DNA bending. In several instances, IHF has been shown to be dispensable for σ^54^-dependent regulation; however, in these cases, DNA bending is induced by stretches of A/T-rich sequences between σ^54^- and EBP-binding elements [35], [41]. No A/T-rich region that may induce natural curvature of DNA is found between the DVU2106- and the σ^54^-binding sites of *orp1* and *orp2* (Figure S1), an observation that may explain the important requirement for IHF for the regulation of the *orp1* and *orp2* operons. Such drastic effect was observed for the TodS-TodT two-component regulatory system from *P. putida* in which the absence of IHF causes a 8-fold decrease of the *todX* promoter expression compared to the wild-type strain [38].

It is noteworthy that these assays have been performed in the heterologous host *E. coli*. The fact that the absence of the *E. coli* IHF causes a decreased in expression of the *orp1* and *orp2* promoters demonstrates that despite the weak homology between the *E. coli* and DvH proteins, they are functionally similar. We therefore took advantage of this observation to (i) use the purified *E. coli* IHF protein for *in vitro* studies and (ii) to identify the DvH functional IHF heterodimer in reconstitution experiments in the *E. coli* Δ*ihfA* strain.

The *E. coli* IHF protein was purified and electrophoretic mobility shift experiments using *orp1* and *orp2* promoter probes showed that, as expected from the IHF requirement for transcriptional activation, IHF binds to both promoters. We therefore went further by identifying the IHF binding sequences within the *orp1* and *orp2* promoters using a combination of computational and mutational analyses coupled to mobility shift and promoters-*lacZ* transcription reporter fusion assays. Bioinformatic analyses revealed the presence of two putative IHF boxes upstream the *orp1* σ^54^ promoter and one upstream the *orp2* σ^54^ promoter. We showed that IHF binds the *orp1* IHF2 and the *orp2* IHF binding sequences. The results from the transcriptional reporter fusions were consistent with these data as alteration within the *orp1* IHF1 site has no effect on the expression level, whereas alterations within the *orp1* IHF2 or *orp2* IHF sites decrease the expression to levels comparable to that of the wild-type promoters in the Δ*ihfA* strain. Therefore, the *orp1* IHF2 and *orp2* IHF binding site are required for IHF-mediated activation of the *orp1* and *orp2* promoters respectively. It is worth to note that *orp1* expression is mediated by the IHF2 site and not IHF1. This result is, at first, surprising because comparison of these sequences with the *E. coli* IHF binding consensus motif shows that the *orp1* IHF2 site is less conserved than IHF1 (67% and 78% respectively, Figure S3) and the 5′ of the IHF1 sequence is 100% identical to the reported consensus (WATCAR) whereas the IHF2 sequence is less conserved in this region (Figure S3).

**Figure 8:**
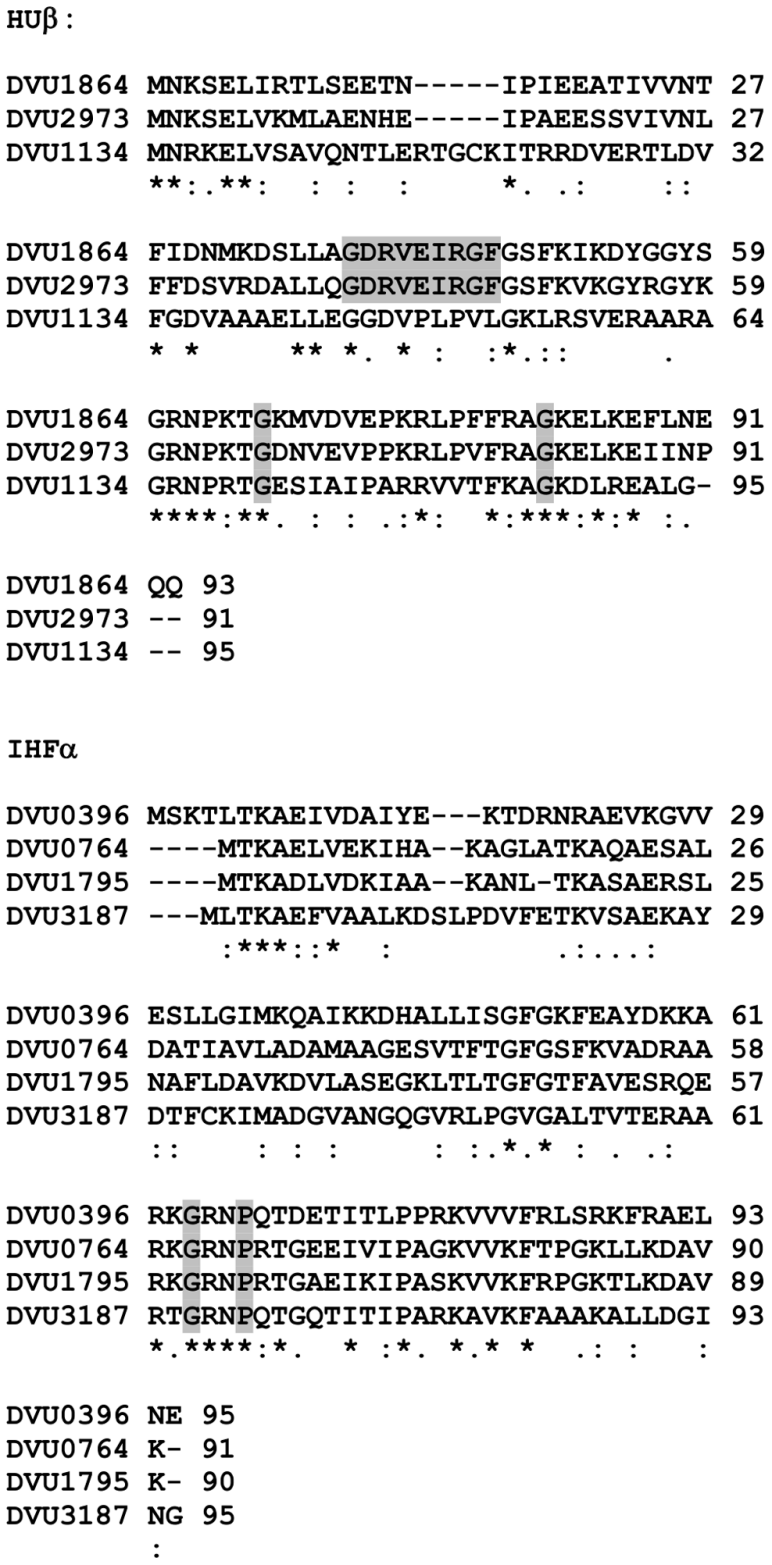
Protein sequence alignment of putative IHFβ and IHFα subunits from DvH. The figure shows the sequence of DVU1864 aligned with the sequences of the two putative IHFβ from DvH, DVU1864 and DVU2973 and the sequence of DVU0396 (IHFα) aligned with the sequences of three putative IHFα from DvH using ClustalW (http://www.ebi.ac.uk). Highlighted residues correspond to crucial amino-acids for IHF function in *E. coli*.

An *in vivo* reconstitution approach in *E. coli* allowed us to identify DVU0396 (IHFα) and DVU1864 (IHFβ) as a functionally active heterodimer able to fully complement the absence of the *E. coli* IHFα subunit. By contrast, DVU0396 alone is unable to complement for the absence of the *E. coli* IHFα subunit and to functionally interact with the *E. coli* IHFβ contrarily to what has been described for *P. putida* IHFα [42]. This could be explained by the difference between the similarities of *E. coli* IHFα and DvH DVU0396 (32%) or *P. putida* IHFα (86%). The fact that DVU0396 and DVU1864 assemble a functionally active heterodimer is in agreement with recent results demonstrating that DVU1864 interacts preferentially with DVU0396 in pull-down experiments [36]. While expression of the *orp* operons is on the same order of magnitude in the presence of *E. coli* IHF and DvH DVU0396/DVU1864 heterodimer, it will be interesting in the future to test DVU0396/DVU1864 binding on the *orp* promoters by EMSA. Experiments are currently underway to purify the DvH DVU0396/DVU1864 complex. While DVU0396 is active in *E. coli*, the absence of DVU0396 does not significantly impact the expression of the *orp* operon genes in DvH as shown by quantitative RT-PCR. One hypothesis to explain this discrepancy is the large number of HU and IHF proteins in DvH that might have redundant functions. By contrast to the *E. coli* situation, DvH appears to lack the diversity of nucleoid-associated proteins such as Fis or HNS but seems to encode several copies of HU and IHF proteins. DvH genome encodes up to eight DNA-binding HU/IHF proteins belonging to the COG776 family. Among these, two are annotated as encoding IHFβ-like proteins (DVU1864 and DVU2973, which are 65% identical) and one, DVU1134, is annotated as a true DNA-binding HUβ protein. Whereas DVU1864 and DVU2973 contains a central region extremely well conserved shown to be essential for IHF activity and specificity in *E. coli* [42], [43], this central region in DVU1134 is less conserved (Figure 8). Three other HU-like proteins (DVU0764, DVU1795, DVU3187) exhibit 25–31% sequence identity with DVU0396. All three proteins possess the G62 residue required for IHF activity and possess one additional residue P65 known to be crucial determinants of IHF specificity in *E. coli* [43]. Such redundancy in genes encoding HU/IHF proteins has not been observed in other organisms studied to date. Therefore, although the DVU0396-DVU1864 combination is functionally active in *E. coli* one may hypothesize that distinct homodimers or heterodimers involving different combinations will be used in DvH as previously described in other organisms [21], [22], [23], [24], and therefore the *DVU0396* mutation will not be sufficient to observe an effect on *orp* gene expression in DvH. This hypothesis is strengthened by pull-down experiments demonstrating that DVU1864, while interacting preferentially with DVU0396, is also able to interact with DVU2973, while this later also interacts with DVU0396 and DVU0764 [37]. Further experiments are therefore needed to establish the role of each HU/IHF proteins and identify all functional IHF complexes coexisting in DvH. It would be also interesting to know whether this atypical gene redundancy is linked to the large number of putative EBPs observed in DvH [6].

**Figure 9:**
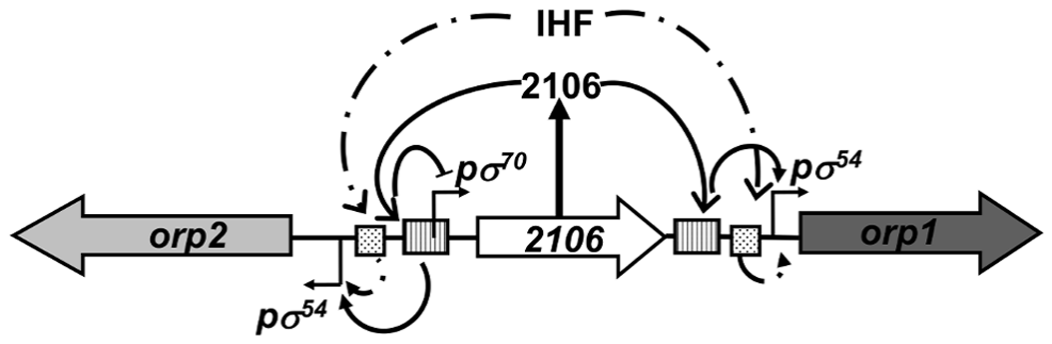
Schematic representation of the regulatory mechanisms of the *orp* gene cluster in DvH. The positions of the σ^54^ and σ^70^ are indicated by bent arrows, the DVU2106-binding sequences are indicated by hatched rectangles and IHF-binding sequences are indicated by blotted rectangles. The DVU2106 transcriptional regulator and IHF play a positive role in the expression of the σ^54^-dependent *orp1* and *orp2* operons. DVU2106 exerts a negative retrocontrol on its own σ^70^-dependent expression.

We recently proposed a model on the regulation of the *orp* operons in which the σ^54^-transcriptional regulator DVU2106 interacts with specific palindromic sequences and collaborates with σ^54^ to activate and synchronize the transcription of the two divergent *orp* operons [6]. DVU2106 is under the control of the housekeeping σ^70^ factor but the overlap between the σ^70^ promoter and the DVU2106-binding sequence creates a negative regulatory loop [6]. With the data presented here, we implemented this model by adding a new player in the game, IHF (Figure 9). We showed that IHF is required for full activity of the *orp1* and *orp2* promoter. As previously suggested, since IHF facilitates DNA bending but does not influence directly the formation of the open complex, its role in σ^54^-dependent systems has to be considered as a co-regulator [44]. In this context, we demonstrated that IHF binds to specific sequences, closely related to the *E. coli* IHF-binding consensus motif, located between the *orp1* and *orp2* σ^54^-promoters and DVU2106 palindromic sites, to co-regulate and orchestrate the simultaneous expression of the divergent *orp* operons. In contrast no effect of IHF was observed on the σ^70^-dependent DVU2106 expression.

## Supporting Information

Figure S1 **Sequences of** ***orp1*** **(A) and** ***orp2*** **(B) promoter regions.** The indentified σ^54^ promoters of *orp1* and *orp2* are boxed in solid lines. The transcriptional start point of both operons is indicated by bent arrows. The stop codon and the start codon are in italics and underlined, and the shine-Dalgarno are indicated in bold. The two palindromic binding sites of the σ^54^ transcription activator, DVU2106, are boxed in dotted lines. The sequences of the putative IHF-binding sites are in bold and underlined: the *orp1* promoter contains two putative IHF-binding sites and one putative IHF-binding sequence was found in the *orp2* promoter.
(PDF)

Figure S2 **Sequences of the different IHF sites and its variants.** Mutated bases are indicated in bold for each mutant variant. These sequences are aligned with the IHF-binding consensus of *E.coli*.
(PDF)

Figure S3 **Sequence alignment of each** ***Dv*** **H IHF-binding site with IHF-binding site consensus sequences of** ***E.coli*** **(A) and** ***P.putida*** **(B).**
(PDF)

Figure S4 **Determination of the consensus sequence of IHF-binding site from the two functional IHF-binding sites in** ***orp*** **promoters of** ***Dv*** **H.** This consensus sequence is aligned with the IHF-binding site consensus sequence of *E.coli* and *P.putida*.
(PDF)

Table S1 **Bacterial strains and plasmids used in this study.**
(PDF)

Table S2 **Primers used in this study.**
(PDF)
