# Supplementary material for: IHF Is Required for the Transcriptional Regulation of the Desulfovibrio vulgaris Hildenborough orp Operons
Source: PLoS One. 2014 Jan 21;9(1):e86507. doi: 10.1371/journal.pone.0086507 (PMC3897727; doi:10.1371/journal.pone.0086507)
Supplement: Figure S1 — Sequences of orp1 (A) and orp2 (B) promoter regions. The indentified σ54 promoters of orp1 and orp2 are boxed in solid lines. The transcriptional start point of both operons is indicated by bent arrows. The stop codon and the start codon are in italics and underlined, and the shine-Dalgarno are indicated in bold. The two palindromic binding sites of the σ54 transcription activator, DVU2106, are boxed in dotted lines. The sequences of the putative IHF-binding sites are in bold and underlined: the orp1 promoter contains two putative IHF-binding sites and one putative IHF-binding sequence was found in the orp2 promoter. (PDF) [file pone.0086507.s001.pdf]

A. *orp1* promoter region :

GGCGACACGAAA*TGA*CGTAGCCGGTCGACGCCATTCCCGGCGCACCCTGCGC
CCTAACGCCGCCTCTCAGTCCCGACAGACCTGTCTGCGCACCCCGTGTAGCCA
GTCTGACCACACCTCGCCGCGCTGGCCTCCATGCCGGTCTCCAAGACCGTTAT
CCCCTGCCCGACGCCTTGGCGGCTATCCCTGCCAGCCCGTCTCCCCCTGACAT
AGGGCGTGTTTGCGCCCACATGGCCACGACAAGGCGCATTTACGCC**CATCA**
CAAGCTCGACGTTCCACGCAAAAAGCAACAACC**AATCAGAATAAA**AGCATTTCT
TTTCACGGCATCATTCCTGCTTCGGAGTGGTC**G**GTACCG**GAGGTA**CGCCACGC
C*ATG*ATCGTCCATGC

+1

B. *orp2* promoter region :

GTTTCGCAGGGGATGTCTCGGGGCAGGGC*CAT*ATGATCCTCCGTGTGAAAGTCT
CTATCATGTGGGCGCATTTTGCGCCCTATTTTTCTTGGGCGTGCATGTGTGTTGG
GAA**AAGATGTTTGATT**ACGATGTATTGGAGTCGTGGAACGGAACGTGCTTCGTC
CGTG**G**CAAACGCTGCATACGCGGCGG**AAGGAGC**ACAT*ATG*CCATACGGAGATG

+1

Figure S1: Sequences of *orp1* (A) and *orp2* (B) promoter regions. The indentified σ^{54} promoters of *orp1* and *orp2* are boxed in solid lines. The transcriptional start point of both operons is indicated by bent arrows. The stop codon and the start codon are in italics and underlined, and the shine-Dalgarno are indicated in bold. The two palindromic binding sites of the σ^{54} transcription activator, DVU2106, are boxed in dotted lines. The sequences of the putative IHF-binding sites are in bold and underlined: the *orp1* promoter contains two putative IHF-binding sites and one putative IHF-binding sequence was found in the *orp2* promoter.
