# Supplementary material for: IHF Is Required for the Transcriptional Regulation of the Desulfovibrio vulgaris Hildenborough orp Operons
Source: PLoS One. 2014 Jan 21;9(1):e86507. doi: 10.1371/journal.pone.0086507 (PMC3897727; doi:10.1371/journal.pone.0086507)
Supplement: Figure S2 — Sequences of the different IHF sites and its variants. Mutated bases are indicated in bold for each mutant variant. These sequences are aligned with the IHF-binding consensus of E.coli. (PDF) [file pone.0086507.s002.pdf]

E. Coli IHF site	WATCAANNNNTTR
orp1 IHF1	AATCAGAATAAAA
	*******
orp1 IHF1-mut	**GGG**CAGAATA**CCC**
E. Coli IHF site	WATCAANNNNTTR
orp1 IHF2	CATCACAAGCTCG
	* ****** *
orp1 IHF2-mut	C**GGG**ACAAGC**C**C**C**
E. Coli IHF site	WATCAANNNNTTR
orp2 IHF	AATCAAACATCTT
	* ********
orp2 IHF-mut	A**CC**CAAACATC**GG**

Figure S2: Sequences of the different IHF sites and its variants. Mutated bases are indicated in bold for each mutant variant. These sequences are aligned with the IHF-binding consensus of *E.coli*.
