# Supplementary material for: IHF Is Required for the Transcriptional Regulation of the Desulfovibrio vulgaris Hildenborough orp Operons
Source: PLoS One. 2014 Jan 21;9(1):e86507. doi: 10.1371/journal.pone.0086507 (PMC3897727; doi:10.1371/journal.pone.0086507)
Supplement: Figure S3 — Sequence alignment of each Dv H IHF-binding site with IHF-binding site consensus sequences of E.coli (A) and P.putida (B). (PDF) [file pone.0086507.s003.pdf]

A

```
E.coli        WATCARxxxxTTR
              ******     *
orp1 IHF1     AATCAGAATAAAA

E.coli        WATCARxxxxTTR
               ****    * *
orp1 IHF2     CATCACAAGCTCG

E.coli        WATCARxxxxTTR
              ******    *
orp2 IHF      AATCAAACATCTT
```

B

```
P.putida      WWWCARxxxxWTR
              ******   * *
orp1 IHF1     AATCAGAATAAAA

P.putida      WWWCARxxxxWTR
               ****    * *
orp1 IHF2     CATCACAAGCTCG

P.putida      WWWCARxxxxWTR
              **   *   **
orp2 IHF      AAGATGTTTGATT
```

Figure S3: sequence alignment of each *Dv*H IHF-binding site with IHF-binding site consensus sequences of *E.coli* (A) and *P.putida* (B).
