# Supplementary material for: IHF Is Required for the Transcriptional Regulation of the Desulfovibrio vulgaris Hildenborough orp Operons
Source: PLoS One. 2014 Jan 21;9(1):e86507. doi: 10.1371/journal.pone.0086507 (PMC3897727; doi:10.1371/journal.pone.0086507)
Supplement: Figure S4 — Determination of the consensus sequence of IHF-binding site from the two functional IHF-binding sites in orp promoters of Dv H. This consensus sequence is aligned with the IHF-binding site consensus sequence of E.coli and P.putida. (PDF) [file pone.0086507.s004.pdf]

orp1' site2	CATCACAAGCTCG
orp2'	AATCAAACATCTT
*Dv*H consensus	XATCAXAXRXXXX
E.Coli consensus	WATCARxxxxTTR
P.Putida consensus	WWWCARxxxxWTR

Figure S4: Determination of the consensus sequence of IHF-binding site from the two functional IHF-binding sites in *orp* promoters of *Dv*H. This consensus sequence is aligned with the IHF-binding site consensus sequence of *E.coli* and *P.putida*.
