# Supplementary material for: IHF Is Required for the Transcriptional Regulation of the Desulfovibrio vulgaris Hildenborough orp Operons
Source: PLoS One. 2014 Jan 21;9(1):e86507. doi: 10.1371/journal.pone.0086507 (PMC3897727; doi:10.1371/journal.pone.0086507)
Supplement: Table S1 — Bacterial strains and plasmids used in this study. (PDF) [file pone.0086507.s005.pdf]

Table S1. Bacterial strains and plasmids used in this study.

Strains or plasmids	Characteristics	Reference or source
***E.coli* strains**		
DH5α		
W3110-*lacZ*	*rrnD-rrnE, rph-1, ΔlacZ*	Fiévet *et al* (2011)
W3110-*lacZ ihf*	*rrnD-rrnE, rph-1, ΔlacZ, Δihf*	Baba *et al* (2006)
***Desulfovibrio vulgaris* Hildenborough strain**	Wild-type strain	Postgate (1984)
*Dv*H Δ*ihfα*	*ihfα* gene replaced by Cm^R gene	This study
**Plasmids**		
pOK12	Cloning vector, P15A replicon, Kan^R	Viera and Messing (1992)
pOK12-2106	Contains the DVU2106 gene fused to an N-terminal FLAG tag in the pOK12 vector	Fiévet *et al* (2011)
pT7.5	Cloning vector, ColE1 replicon, Amp^R	Tabor and Richardson (1985)
pT7.5-*porp2::lacZ*	Contains the promoter region of *orp2* fused to the *lacZ* gene	Fiévet *et al* (2011)
pT7.5-*pDVU2106::lacZ*	Contains the promoter region of *DVU2106* fused to the *lacZ* gene	Fiévet *et al* (2011)
pT7.5-*porp1::lacZ*	Contains the promoter region of *orp1* fused to the *lacZ* gene	Fiévet *et al* (2011)
pT7.5-*porp2mut::lacZ*	Contains the promoter region of *orp2* mutated for the putative IHF binding site fused to the *lacZ* gene	This study
pT7.5-*pDVU2106mut::lacZ*	Contains the promoter region of *DVU2106* mutated for the putative IHF binding site fused to the *lacZ* gene	This study
pT7.5-*porp1mut1::lacZ*	Contains the promoter region of *orp1* mutated for the putative IHF binding site 1 fused to the *lacZ* gene	This study
pT7.5-*porp1mut2::lacZ*	Contains the promoter region of *orp1* mutated for the putative IHF binding site 2 fused to the *lacZ* gene	This study
pT7.5-*porp1mut::lacZ*	Contains the promoter region of *orp1* mutated for the two putative IHF binding sites fused to the *lacZ* gene	This study
porc-IHF		Murtin *et al* (1998)
pNot19	Cloning vector pUC19. NdeI site replaced by a NotI site	
pDel	pNot19 with Cm^R gene cloned into the SpeI and MfeI sites	This Study
pDel*ihfα*	pDel with upstream and downstream regions of *ihfα* gene (DVU0396) cloned into the AscI/SpeI and MfeI/BglII sites respectively	This study

- Fiévet, A., My, L., Cascales, E., Ansaldi, M., Pauleta, SR., Moura, I., Dermoun, Z., Bernard, CS., Dolla, A., and Aubert, C (2011) The anaerobe-specific orange protein complex of Desulfovibrio vulgaris Hildenborough is encoded by two divergent operons coregulated by σ^{54} and a cognate transcriptional regulator. J. Bacteriol. 193:3207-3219.
- Baba T, Ara T, Hasegawa M, Takai Y, Okumura Y, Baba M, Datsenko KA, Tomita M, Wanner BL, Mori H. Construction of Escherichia coli K-12 in-frame, single-gene knockout mutants: the Keio collection. (2006) Mol Syst Biol.;2:2006.0008. Epub 2006 Feb 21.
- Postgate, J.R (1984) in The Sulphate-reducing bacteria, 2nd ed; pp12-13, Cambridge University Press, Cambridge, UK.
- Vieira, J., and Messing, J. (1991) New pUC-derived cloning vectors with different selectable markers and DNA replication origins. *Gene.* 100: 189-94.
- Tabor, S., and Richardson, C.C. (1985) A bacteriophage T7 RNA polymerase/promoter system for controlled exclusive expression of specific genes. *Proc Natl Acad Sci U S A.* 82: 1074-8.
- Murtin C, Engelhorn M, Geiselmann J, Boccard F (1998) A quantitative UV laser footprinting analysis of the interaction of IHF with specific binding sites: re-evaluation of the effective concentration of IHF in the cell. J Mol Biol. Dec 11;284(4):949-61.
