# Supplementary material for: IHF Is Required for the Transcriptional Regulation of the Desulfovibrio vulgaris Hildenborough orp Operons
Source: PLoS One. 2014 Jan 21;9(1):e86507. doi: 10.1371/journal.pone.0086507 (PMC3897727; doi:10.1371/journal.pone.0086507)
Supplement: Table S2 — Primers used in this study. (PDF) [file pone.0086507.s006.pdf]

Table S2. Primers used in this study.

Primer	Sequence (5'-3')
**For transcriptional fusions**	
Prom2105	5'-CTCACTATAGGGAGACCGGAATTCGAGCTCCTATGATCCGTGTGAAAGTCTCTATCATGTG-3'
Prom2105_comp	5'-GGTCGACTCTAGAGGATCCCCGGGCCATATGTGCTCCTTCCGCCGCGTATGC-3'
Prom2106	5'-CTCACTATAGGGAGACCGGAATTCGAGCTCCATATGTGCTCCTTCCGCCGCGTATGC-3'
Prom2106_comp	5'-GGTCGACTCTAGAGGATCCCCGGGCCATATGATCCTCCGTGTGAAAGTCTCTATCATGTG-3'
Prom2107	5'-CTCACTATAGGGAGACCGGAATTCGAGCTCCGTAGCCGGTCGACGCCATTCCCG-3'
Prom2107_comp	5'-GGTCGACTCTAGAGGATCCCCGGGCCATGGCGTGGCGTACCTCCGGTACC-3'
pOK2106	5'-CGAGATCTATCGATGCATGCCATGGACTACAAAGACGACGATGACAAGGGGGCCCTGCCCCGAGACATCCCCTGCG-3'
pOK_comp	5'-GAAGCTTCGAATTCGAGCTCCCGGGTATCATTTCGTGTCGCCACGTTGCAGGATG-3'
**For IHF binding site directed mutagenesis**	
2105IHFmut-dir	5'-CGACTCCAATACATCGTACCCAAACATCGGTTCCCAACACACATGCAC6-3'
2105IHFmut-rev	5'-GTGCATGTGTGTTGGGAACCGATGTTTGGGTACGATGTATTGGAGTCG-3'
2107IHFmut1-dir	5'-CACGCAAAAAGCAACAACCGGGCAGAATACCCGCATTTCTTTTCACGGCATC-3'
2107IHFmut1-rev	5'-GATGCCGTGAAAAGAAATGCGGGTATTCTGCCCGGTTGTTGCTTTTTGCGTG-3'
2107IHFmut2-dir	5'-CAAGGCGCATTTTACGCCCGGGACAAGCCCCACGTTCCACGCAAAAAGC-3'
2107IHFmut2-rev	5'-GCTTTTTGCGTGGAACGTGGGGCTTGTCCCGGGCGTAAAATGCGCCTTG-3'
**For gel mobility shift assay**	
Prom2105	5'-CTCACTATAGGGAGACCGGAATTCGAGCTCCTATGATCCGTGTGAAAGTCTCTATCATGTG-3'
Prom2105_comp	5'-GGTCGACTCTAGAGGATCCCCGGGCCATATGTGCTCCTTCCGCCGCGTATGC-3'
Prom2106	5'-CTCACTATAGGGAGACCGGAATTCGAGCTCCATATGTGCTCCTTCCGCCGCGTATGC-3'
Prom2106_comp	5'-GGTCGACTCTAGAGGATCCCCGGGCCATATGATCCTCCGTGTGAAAGTCTCTATCATGTG-3'
Prom2107	5'-CTCACTATAGGGAGACCGGAATTCGAGCTCCGTAGCCGGTCGACGCCATTCCCG-3'
Prom2107_comp	5'-GGTCGACTCTAGAGGATCCCCGGGCCATGGCGTGGCGTACCTCCGGTACC-3'
**For *Desulfovibrio vulgaris* Hildenborough Δ*ihf*α mutant**	
DVU0395AscI_dir	5'-GTATGGCGCGCCGGAGAACCCCATGTCCCAGAG-3'

CterDVU0395-SpeI	5'-TAATCTAACTAGTCTAGTCCATGCGTTCCCTGATGG-3'
DVU0396MefI_dir	5'-TCATCAATTGCATAGCCCTATTTCAGGACG-3'
DVU0396BglII_rev	5'-CCATAGATCTGCTGAGTGAAGCCGAGCGGA-3'
**For verified Desulfovibrio vulgaris Hildenborough Dihfa strain**	
NterDVU0397-dir	5'-ATGCCAAACACCATCCTGCG-3'
NterDVU0394-dir	5'-TCCGGATGGGGAAAAATCAT-3'
**For real-time PCR analysis**	
IHF_right	5'-GTCATAGGCCTCGAACTTGC-3'
IHF_left	5'-GACCAAAGCGGAAATAGTCG-3'
DVU2104_left b	5'-GTGGACTGTGATGTCGAGGAG-3'
DVU2104_right b	5'-AGACGAGTTCACAGAGTCCACA-3'
DVU2106_left	5'-CGACCTCTACTATCGGCTGAAT-3'
DVU2106_right	5'-ACAGGATGAAGGCATATTCGAT-3'
DVU2108_up	5'-ATTCAGGCTGCCCAGATGG-3'
DVU2108_down+	5'-GTGTCGGCAGGGCGGAGT-3'
